# Effect of Multi-Microbial Probiotic Formulation Bokashi on Pro- and Anti-Inflammatory Cytokines Profile in the Serum, Colostrum and Milk of Sows, and in a Culture of Polymorphonuclear Cells Isolated from Colostrum

**DOI:** 10.1007/s12602-017-9380-9

**Published:** 2018-01-05

**Authors:** Ewa Laskowska, Łukasz Jarosz, Zbigniew Grądzki

**Affiliations:** Department of Epizootiology and Clinic of Infectious Diseases, Faculty of Veterinary Medicine, University of Life Sciences in Lublin, Głęboka 30, 20-612 Lublin, Poland

**Keywords:** Effective microorganisms, Colostrums and milk, Immunoglobulin and cytokine concentration, ConA stimulation of PMC, Pigs

## Abstract

The use of probiotics in sows during pregnancy and lactation and their impact on the quality of colostrum and milk, as well as the health conditions of their offspring during the rearing period, are currently gaining the attention of researchers. The aim of the study was to determine the effect of Bokashi formulation on the concentrations of pro- and anti-inflammatory cytokines in the serum of sows during pregnancy, in their colostrum and milk, and in a culture of Con-A-stimulated polymorphonuclear cells (PMNs) isolated from the colostrum. The study was conducted on 60 sows aged 2–4 years. EM Bokashi were added to the sows’ feed. The material for the study consisted of peripheral blood, colostrum, and milk. Blood samples were collected from the sows on days 60 and 114 of gestation. Colostrum and milk samples were collected from all sows at 0, 24, 48, 72, 96, 120, 144, and 168 h after parturition. The results indicate that the use of Bokashi as feed additives resulted in increased concentrations of pro-inflammatory cytokines TNF-α and IL-6, which increase the protective capacity of the colostrum by stimulating cellular immune mechanisms protecting the sow and neonates against infection. At the same time, the increased concentrations of cytokines IL-4, IL-10, TGF-β, and of immunoglobulins in the colostrum and milk from sows in the experimental group demonstrate the immunoregulatory effect of Bokashi on Th2 cells and may lead to increased expression of regulatory T cells and polarization of the immune response from Th1 to Th2.

## Introduction

Newborn piglets in the initial period of life are exposed to various microbes colonizing their external environment [1]. The high mortality of piglets during this period is most often linked to diarrhea caused by infections with enterotoxigenic strains of *Escherichia coli*, *Salmonella* spp., *Campylobacter* spp., rotaviruses, coronaviruses, and protozoa of the genus *Cryptosporidium* [2]. During this period, the immune potential of piglets to infections is primarily built up by means of passive immunization involving the colostrum, which contains immunoglobulins [3]. In addition to antibodies, the colostrum of sows contains other substances that affect the development of the immune system of piglets and exert an immunomodulatory effect [4]. These include cytokines, which are involved in systemic defense processes through induction of a Th1 or Th2 immune response, dependent on the infectious agent [5]. These proteins have been shown to exert a local effect on the intestinal mucosa and to penetrate the circulatory system, affecting colonization of the gastrointestinal tract of piglets by commensal microbiota at a later stage of life [6]. Colostrum quality and passive protection potential in neonates can be improved by using immunomodulatory compounds, including probiotics, in the diet of sows during colostrogenesis and lactation [7, 8]. Feed supplementation with probiotics has been shown to affect the gut microflora in sows [8] and to influence the composition of colostrum and milk [9, 10]. The beneficial effect of probiotic bacteria on host defense mechanisms involves regulation of the composition of gut microflora [11]), stimulation of humoral immune response mechanisms [12], and maintenance of homeostasis in the body by ensuring a balance between pro- and anti-inflammatory cytokines [13]. Rautava et al. [9] demonstrated that the use of *Lactobacillus rhamnosus* as a dietary supplement in women promotes production of TGF-β, which affects IgA synthesis in milk. Jang et al. [14] showed that the use of probiotics as feed additives for sows stimulates IgG synthesis and increases the concentration of this immunoglobulin in the colostrum and in the serum of piglets. A positive effect of probiotics on growth, metabolism, and nutrient digestibility in weaning piglets and grower-finisher pigs has been reported as well [15, 16]. From an economic perspective, what is most important is the beneficial effect of probiotics on performance indicators, including increased litter size, litter weight, the quantity of milk produced, and the quality of its composition [17–19].

A variety of probiotic preparations are currently used in pig diets, based on strains of *Lactobacillus*, *Bifidobacterium*, and *Enterococcus* [20]. Probiotic formulations are a broader group of organisms and substances which contribute to intestinal microbial balance and are widely used in numerous fields associated with agriculture and livestock farming [7, 21–23]. The beneficial effect of probiotic preparations on animals is manifested as increased daily weight gain, improved feed digestibility, reduced mortality, and improved health [24–27]. No studies have yet been conducted on the use of Bokashi preparation in sows during pregnancy and lactation and their effect on the immunological quality of the colostrum and milk. The term EM Bokashi^®^ is the name of product invented by manufacturer Greenland Technologia EM, Janowiec, Poland, and describes preparation that contain microbial strains which are not subjected to any technological processing, and it is an indicator of the high quality such products.

The aim of the study was to determine the effect of Bokashi formulation on the concentrations of pro- and anti-inflammatory cytokines in the serum of sows during pregnancy, in their colostrum and milk, and in a culture of Con-A-stimulated PMNs (polymorphonuclear cells) isolated from the colostrum.

## Materials and Methods

### Experimental Animals and Use of Bokashi Preparation

The study was conducted on a private pig breeding farm with a closed production cycle. The farm kept 150 sows aged 2–4 years with a body weight of 150–200 kg. The ‘all in-all out’ principle was followed in the farrowing room. From mating to 100 days of gestation, the sows were kept in individual pens on litter. Two weeks before parturition, they were transferred to the farrowing room, in which the pens had a concrete floor covered with litter. The pregnant and lactating sows were fed individual diets depending on the stage of gestation and the number of piglets being reared. The sows were fed PR-C (full-feed diet for the gestation period) twice daily during pregnancy and PR-L (full-feed diet for the lactation period) during lactation, with constant access to water. From the start of its operation, the farm had been free of contagious diseases, including Aujeszky’s disease, porcine reproductive and respiratory syndrome (PRRS), pleuropneumonia, and mycoplasmosis, as confirmed by screening tests performed at the National Veterinary Research Institute in Puławy. Pregnant sows were regularly vaccinated against parvovirus and erysipeloid of Rosenbach.

All procedures used during the research were approved by the Local Ethics Committee for Animal Testing at the University of Life Sciences in Lublin, Poland (approval number 55/2013, 15 October 2013). The study was conducted on 60 female crossbred sows (Polish Large White × Polish Landrace) in their second parity with an initial body weight of 160–180 kg, at the age of 2 years, all born on the same farm. The trial comprised the first 60 sows (Polish Large White × Polish Landrace) in a system of continuous farrowing. The sows were assigned to two groups, with 30 sows in each group: I: control group and II: experimental group. Sows were included in the study on the basis of body condition score (BCS) according to Charette et al. [28]; the BSC of the sows in both groups was 3.

The control and experimental groups were housed in separate pig houses, with identical building and environmental conditions, in order to prevent probiotic (Bokashi) cross-contamination. The size, temperature, humidity, and hygiene conditions of the pens where the sows were kept were identical for the two groups. Each sow was housed in an individual pen (2200 cm × 2000 cm) during the gestation and lactation period. The lighting program was 16 h of light and 8 h of darkness. The housing area was kept at 20–21.5 °C under humidity of 60–65%, and the ventilation rate was 0.567–0.598 m^3^/min per sow. No antibiotic growth promoters or antibiotic treatment were used during the entire experimental period. Individual feeding of the sows was ensured by specially installed troughs; water was supplied from individual nipple drinkers. All sows were fed the same basal diet during the experiment (Table 1). From fertilization to the 90th day of gestation, the sows received feed for pregnant sows (PR-C), and from the 90th day of gestation to the 28th day of lactation, they received feed for lactating sows (PR-L). The diets were formulated to meet or exceed the nutrient requirement recommendations of the NRC (1998) [29].

**Table 1:** Ingredients and nutritive value of the sow diets (as-fed basis)

Ingredient (g)	Pregnant sow (PR-C)	Lactating sow (PR-L)
Barley	36	33
Wheat	29.50	34
Oats	15	10
Triticale	10	–
Soybean meal over 46% HP	3	12
Fermented rapeseed meal	4	4
Fermented soybean meal	–	2
Soybean oil	–	1
Ultramix L.K. Hi Milk 4%	–	4
Ultramix L.P. Hi Breed 2.5%	2.50	–
Nutritive value		
Metabolizable energy, MJ/kg	12.60	12.8
Crude protein, g	132	168
Dry matter, g	823	812
Lysine, g	5.73	9.53
Methionine,g	2.17	2.92
Methionine + cystine, g	4.96	5.87
Threonine, g	4.32	6.98
Tryptophan, g	1.48	2.00
Digestible lysine, g	0.472	0.972
Total phosphorus, g	5.08	6.58
Digestible phosphorus, g	2.87	4.31
Calcium, g	7.02	9.91
Sodium, g	1.75	2.38
Fiber, g	57.8	51.3
Raw fat, g	21.9	30.1
Vitamin A, IU	13,000	12,000
Vitamin D_3_, IU	2000	2000
Vitamin E, mg	117	165

The sows in group I, the control, were fed from mating to weaning on standard feed without a probiotic supplement. For the sows in group II, the experimental group, probiotic in the form of the preparation EM Bokashi^®^ was added to the basal feed in the amount of 10 kg/t of feed, from mating to weaning. The probiotic EM Bokashi^®^ used in the experiment is manufactured by the commercial company Greenland Technologia EM, Janowiec, Poland, and contains mixed microorganisms; see Table 2. The feed for the control and experimental groups was prepared daily during the entire experiment. Throughout the experimental period, Bokashi preparation was tested once a month in the national reference laboratory of the Department of Hygiene of Animal Feeding stuffs of the National Veterinary Research Institute in Puławy. The viability of probiotic bacterial cells and their content per gram of the product (CFU/g) was tested to ensure that the experimental conditions were the same throughout the experiment. Furthermore, the company manufacturing Bokashi preparation evaluated the viability of probiotic bacterial cells and their content per gram of the product in their laboratory, thereby guaranteeing that the product used in the experimental group of sows was of the same quality.

**Table 2:** EM Carbon Bokashi composition

	Microbial composition	Strain number	Content per gram of product
1.	*Sacharomyces cerevisiae*	Y200007	5 × 10^4^ CFU/g
2.	*Lactobacillus casei*	*ATCC 7469*	5 × 10^8^ CFU/g
3.	*Lactobacillus plantarum*	*ATCC 8014*	5 × 10^8^ CFU/g
4.	*Enterococcus faecalis*	UC-100 (CGMCC No.1.0130)	2.5 × 10^6^ CFU/g
5.	*Enterococcus faecium*	NCIMB SF68	5 × 10^9^ CFU/g
6.	*Bifidobacterium bifidum*	ATCC 29521	5 × 10^8^ CFU/g
7.	*Bifidobacterium pseudolongum*	ATCC 25526	5 × 10^8^ CFU/g
8.	*Bacillus licheniformis*	DSM 5749	4 × 10^9^ CFU/g
9.	*Bacillus cereus var. toyoi*	NCIMB 40112	4 × 10^9^ CFU/g
10.	*Bacillus subtilis*	MA139	4 × 10^11^ CFU/g
11.	*Clostridium butyricum*	MIYAIRI 588 (CBM588)	1 × 10^8^ CFU/g

### Blood Sample Collection

The material for the study consisted of peripheral blood collected from the external jugular vein into serum separation tubes with a clot activator (Medlab Products, Poland). Blood samples were collected from all sows in groups I and II on days 60 and 114 of gestation. The samples were transported chilled to the laboratory at + 4 to + 8 °C for no longer than 1 h. The blood collected into serum separation tubes with a clot activator was centrifuged at room temperature (20–22 °C) for 15 min at 1000*g*, and the serum obtained was apportioned and stored for further study at − 80 °C.

### Collection and Preparation of Colostrum and Milk Samples

Ten-milliliter samples of colostrum and milk were collected into sterile plastic test tubes (Medlab Products, Poland). Sows were given 1 mL of oxytocin (1 U/mL) to stimulate milk release. Milk was manually collected from all functional teats after swabbing with alcohol. Colostrum and milk samples were collected from all sows in groups I and II at 0, 24, 48, 72, 96, 120, 144, and 168 h after parturition, beginning with the first piglet born. The samples were transported chilled to the laboratory at + 4 to + 8 °C for no longer than 1 h. Immediately after delivery to the laboratory, 5 ml of the material was designated for determination of cytokine concentration and the remainder for isolation of PMCs. Five-milliliter volumes of the samples were centrifuged at 600×*g* for 15 min at 15 °C. Then, the fat layer was separated and removed and the supernatant was apportioned and stored for further study at − 80 °C.

### Isolation and Culture of PMNs from Colostrum

Colostrum was diluted 1:3 in 50-mL conical tubes (BD Falcon, Becton Dickinson) with sterile PBS to decrease viscosity. The diluted colostrum samples were centrifuged (15 min at 600×*g*), the cream layer was removed, and the cell pellets were washed in 20 mL PBS-FCS (Invitrogen, Poland) 3 times at 600×*g* for 10 min at 15 °C before isolation of PMNs. Colostrum PMNs were isolated by Ficoll density centrifugation as described by Le Jan [30]. PMNs were resuspended in complete RPMI medium (Invitrogen, Poland) and plated in a 3-well culture (3 wells/sample) on a 96-well microtiter plate (MTP). For evaluation of cytokine production, PMNs were incubated with 5 μg/ml of concanavalin-A (Con-A) for 72 h in a humidified incubator at 37 °C in 5% CO_2_ atmosphere.

### Cytokine and Immunoglobulin Analysis in Sow Serum, Colostrum, and Milk and in Colostrum PMN Culture Supernatant

ELISA kits specific for porcine IL-2, IL-4, IL-10, IL-6, TNF-α, IFN-γ, TGF-β, IgG, and IgA (USCN Life Science Inc., Wuhan) were used to determine the cytokine levels in the sow serum, colostrum, and milk and in the culture supernatant from PMNs isolated from colostrum after 72 h of incubation, following the manufacturer’s instructions. Each sample was tested in three replicates. The results were expressed as mean and standard deviation (±SEM); values of *p* < 0.05 were regarded as significant.

### Assessment of Production and Breeding Effects

During the experiment, the sows remained under clinical observation, which focused on their feed intake, body weight at farrowing, weight loss during lactation, and lactation length. The number of piglets born and their birth weight was determined as well. Particular attention was paid to the occurrence of gastrointestinal disturbances with diarrhea in the piglets and piglet mortality during the first 7 days of life (Table 4).

### Statistical Analysis

**Fig. 1:**
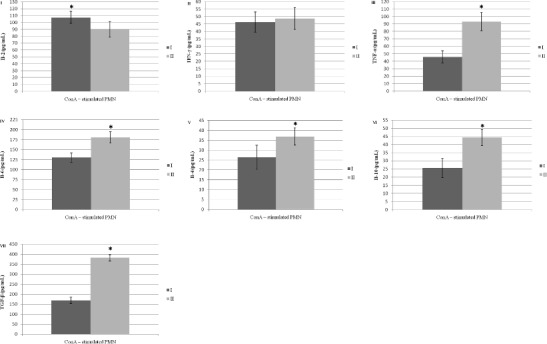
Cytokine production by ConA-stimulated PMNs, isolated from sows colostrum: I–Il-2, II–IFN-γ, III–TNF-α, IV–Il-6, V–Il-4, VI–Il-10, VII–TGF-β. I–control group, II–experimental group. Values are expressed as the mean and standard deviation (α^+^/-SD). Asterisks indicate a significant increase in the parameter (*p < 0.05) between control and experimental group

**Fig. 2:**
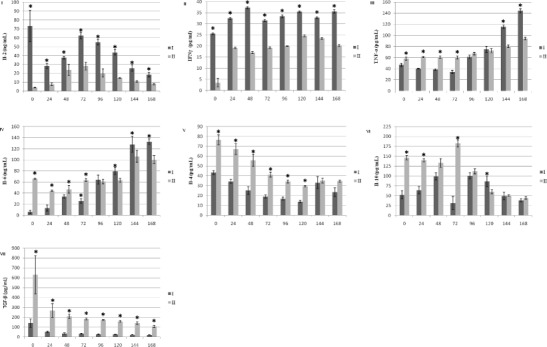
Colostrum and milk concentration of: I–Il-2, II–IFN-γ, III–TNF–α, IV–Il-6, V–IL-4, VI–IL-10, VII–TGF-β in sows. I–control group, II–experimental group. Values are expressed as the mean and standard deviation (α^+^/-SD). Asterisks indicate a significant increase in the parameter (*p < 0.05) between control and experimental group

**Fig. 3:**
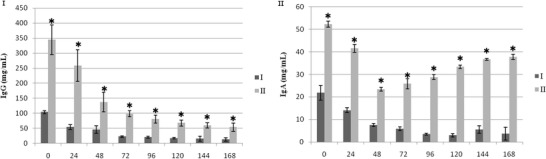
Colostrum and milk concentration of: I–IgG, II–IgA in sows. I–control group, II–experimental group. Values are expressed as the mean and standard deviation (α^+^/-SD). Asterisks indicate a significant increase in the parameter (*p < 0.05) between control and experimental group

The results were analyzed statistically using Statistica 10.0 PL (StatSoft, Krakow, Poland). The analysis included the arithmetic mean and standard deviation (α ± SD). The significance of differences between means obtained for the control and experimental groups of animals was assessed by the nonparametric Mann-Whitney *U* test, and *P* values of less than 0.05 were considered to indicate statistical significance (Table 3 and Figs. 1, 2, 3).

**Table 3:** Serum concentration of Il-2, IFN-γ, TNF-α, Il-6, Il-4, Il-10, TGF-β, IgG, IgA, in sows. Values are expressed as the mean and standard deviation (α+/-SD)

	Day 60		Day 114	
Parameter	I	II	I	II
Il-2 (pg/mL)	73.67 ± 10.1	76.74 ± 9.9	79.62 ± 8.7	84.57 ± 11.1^a^
IFN-γ (pg/mL)	158.24 ± 9.4	164.11 ± 10.6	165.28 ± 10.1	172.04 ± 11.4^a^
TNF-α (pg/mL)	58.96 ± 8.8	63.14 ± 9.2	69.61 ± 8.7	89.43 ± 8.9^a^
Il-6 (pg/mL)	68.87 ± 9.4	82.34 ± 11.1^a^	126.75 ± 8.6	126.96 ± 12.2
Il-4 (pg/mL)	250.92 ± 11.8	750.69 ± 16.5^a^	261.42 ± 9.8	1895.37 ± 24.6^a^
Il-10 (pg/mL)	564.12 ± 16.1	620.68 ± 18.6^a^	539.33 ± 14.2	584.21 ± 16.3^a^
TGF-β (pg/mL)	256.55 ± 21.8	279.02 ± 22.15	331.51 ± 22.6	1244.27 ± 31.7^a^
IgG (mg/mL)	16.91 ± 3.8	18.62 ± 4.2	16.41 ± 2.9	24.99 ± 7.4^a^
IgA (mg/mL)	0.91 ± 0.2	1.01 ± 0.3	1.06 ± 0.3	2.18 ± 0.4^a^

^a^Asterisks indicate a significant increase in the parameter (*p < 0.05) between control and experimental group. I–control group, II–experimental group

## Results

### Evaluation of Serum Concentration of Cytokines and Immunoglobulin in Sows

Analysis of the results for IL-2 concentration in the serum of the sows on days 60 and 114 of the experiment revealed statistically significantly higher values (*p* < 0.05) for this parameter in the experimental group (II) than in the control group (I) on day 114 (Table 3). A similar relationship was noted for the concentrations of IFN-γ and TNF-α. A statistically significant increase (*p* < 0.05) in the serum concentration of IL-6 between the experimental group and the control group was observed in the samples taken on the 60th day of the study. A statistically significant increase (*p* < 0.05) in the concentrations of IL-4 and IL-10 as compared to the control group was observed in both cases in samples taken on days 60 and 114 of the study. Analysis of the concentrations of TGF-β and immunoglobulins G and A in the sow serum showed a statistically significant increase (*p* < 0.05) in their concentrations as compared to the control group (S) on day 114 of the study. Detailed data are shown in Table 3.

### Evaluation of Cytokine Concentrations in the ConA-Stimulated PMN Cultures

The set of graphs in Fig. 1 shows the cytokine production by ConA-stimulated PMNs isolated from sow colostrum. For IL-2, a statistically significantly higher concentration (*p* < 0.05) was observed in the colostrum from the sows in the control group (Fig. 1I). There were no statistically significant differences in the concentration of IFN-γ between the experimental and control groups (Fig. 1II). In the case of TNF-α, IL-6, IL-4, IL-10, and TGF-β, a statistically significant increase (*p* < 0.05) was observed in their concentrations in the colostrum from sows in the experimental group as compared to the control (Figs. 1III, IV, V, VI, VII).

### Evaluation of Cytokine and Immunoglobulin Concentrations in the Colostrum and Milk

The set of graphs in Figs. 2 and 3 shows the colostrum and milk concentrations of IL-2, IL-4, IL-10, IL-6, TNF-α, IFN-γ, TGF-β, IgG, and IgA at 0, 24, 48, 72, 96, 120, 144, and 168 h after parturition. The concentrations of IL-2 and IFN-γ were statistically significantly higher (*p* < 0.05) in the control group than in the experimental group at all sampling times (0–168 h) (Figs. 2I, II). In comparison to the control group, statistically significantly higher (*p* < 0.05) colostrum and milk concentrations of TNF-α were obtained in the experimental group at 0, 24, 48, 72, 144, and 168 h after parturition (Fig. 2III). Figure 2IV shows the concentration of IL-6. As compared to the control group, statistically significantly higher (*p* < 0.05) colostrum and milk concentrations of this cytokine were obtained in the experimental group at 0, 24, 48, 72, 120, 144, and 168 h after parturition. Statistically significantly higher (*p* < 0.05) colostrum and milk concentrations of IL-4 were obtained in the experimental group at 0, 24, 48, 72, 96, and 120 h after parturition as compared to the control (Fig. 2V). Figure 2VI shows the concentration of IL-10. In comparison to the control group, statistically significantly higher (*p* < 0.05) colostrum and milk concentrations of this cytokine were obtained in the experimental group at 0, 24, 72, and 120 h after parturition. Concentrations of TGF-β, IgG, and IgA were statistically significantly higher (*p* < 0.05) in the experimental group than in the control group at all sampling times (0–168 h) (Fig. 2VII and Fig. 3I, II).

### Production and Breeding Evaluation

Compared to the control group in the experimental group, there was a statistically significant increase (*p* < 0.05) in sow weight at farrowing, increase in lactation length, greater number of new born piglets, and higher birth weight of newly born piglets. Compared to the control group, there was also a lower mortality rate of piglets within first 7 days after birth and lower percentage of piglets with diarrhea within 7 days after birth in the experimental group. There were no differences between the control and experimental groups in the feed consumption, but the percentage of sow weight loss (kg) was higher in the control group (Table 4).

**Table 4:** The effect of effective microorganisms (EM) in sow diets on performance parameters (mean and α^+^/-SD)

Parameter	Control group	Experimental group
Sow weight at farrowing, kg	231.6 (22.8)	241.5 (16.4)^a^
Sow weight loss, kg	38.1 (2.9)^a^	25.2 (7.4)
Feed consumption, kg	232.4 (9.5)	238.6 (18.5)
Lactation period, day	26.4 (2.2)	30.1 (3.1)^a^
Number of piglets born	11.2 (2.5)	12.4 (1.5)^a^
Piglet birth weight, kg	1.46 (0.62)	1.68 (0.31)^a^
Mortality in first 7 days, %	7.74 (2.1)	4.84 (1.2)^a^
Diarrhea in pigs in first 7 days %	6.25 (3.2)	3.22 (1.6)^a^

^a^Asterisks indicate a significant increase in the parameter (*p < 0.05) between control and experimental group

## Discussion

One way to improve the immunological quality of colostrum is to modify the diet of sows during pregnancy and lactation by using probiotics as feed additives [19]. The use of probiotics in human and animal diets during pregnancy and lactation has also been shown to increase the immune potential of colostrum and milk and to protect offspring against disease [9, 31–33]. The results of these studies suggest that probiotics used as dietary supplements have an immunomodulatory effect. This is what we found in our experiment using Bokashi preparation as a feed additive for sows during pregnancy and lactation.

Previously published research has shown that probiotics induce production of Th1 pro-inflammatory cytokines while at the same time increasing synthesis of IL-10 and TGF-β, which have anti-inflammatory functions [34]. Such an effect is confirmed by the results of our experiment, in which the use of Bokashi in sows during pregnancy was followed by an increase in the concentration of Th1 cytokines, i.e., IL-2, TNF-α, and IFN-γ, and Th2 cytokines, i.e., IL-4, IL-10 and the immunoregulatory TGF-β. Dahan et al. [35] and Dalmasso et al. [36] showed that the use of S*treptococcus boulardii* as a dietary supplement in humans inhibits the release of pro-inflammatory cytokines such as IL-8, IL-6, and TNF-α in response to infection. The use of *S. boulardii* protects cells in the infection process by inhibiting signal pathways of transcription factor NF-κB, which is responsible for inducing expression of pro-inflammatory mediators, and of mitogen-activated protein kinases (MAPK), thereby reducing production of pro-inflammatory cytokines. Furthermore, *S. boulardii* exerts a unique effect on inflammation through a specific alteration of the migratory behavior of T cells, which accumulate in mesenteric lymph nodes; therefore, *S boulardii* treatment limits infiltration of T helper 1 cells and the inflammatory process. Our observations are consistent with the results of a study by Zhang et al. [37], which demonstrated that *Lactobacillus rhamnosus* used in pigs affects pro- and anti-inflammatory cytokines by regulating their systemic production. Changes in the concentrations of cytokines secreted by Th1 or Th2 cells are associated with altered proportions of lymphocyte subpopulations, with no change in the function of these cells. The high percentage of pro- and anti-inflammatory cytokines shown in our study in the serum of pregnant sows indicates that Bokashi preparation have an immunomodulatory effect on systemic immune processes, manifested in part as improvement in the immunological quality of the colostrum. This is also confirmed by research published by Bailey et al. [38] and Scharek et al. [12], who showed a high percentage of CD25^+^ and CD8^+^ lymphocytes in the intestinal lamina propria of pigs whose feed was supplemented with *Bacillus cereus*. These observations indicate stimulation of an immune response associated with the development of the GALT system and the activation of regulatory T cells. On the other hand, Schierack et al. [39] showed that in sows that did not receive probiotics, the relative numbers of CD21^+^, CD4^+^, and CD8^+^ cells decreased, whereas supplementation of feed with *Bacillus cereus* during pregnancy mainly stimulated the subpopulation of CD21^+^ lymphocytes in blood samples. It should therefore be assumed that stimulation of lymphocyte subpopulations by the specific bacterial antigens contained in Bokashi preparation enhances their proliferation and stimulates the production of regulatory cytokines circulating in the peripheral blood and entering the colostrum and milk. These phenomena improve the function of the sow immune system, protecting the developing fetuses and increasing the immune potential of the colostrum and milk. The stimulatory function of Bokashi preparation is also confirmed by the high serum IgG and IgA antibody concentrations in the sows on day 114 of gestation and in the colostrum and milk in the postpartum period. Assuming that 40% of the IgA and 100% of the IgG present in colostrum originates in the serum of the sow [40], it can be concluded that the high concentrations of these immunoglobulins in the experimental group will enhance passive protection of the piglets against infections in the first few days after birth [40, 41].

In addition to antibodies, colostrum has other protective and immunomodulatory components that protect neonates against pathogenic microbes [42]. Other than immunoglobulins, the main immunomodulatory compounds present in colostrum include cytokines, chemokines, and growth factors [43]. The cytokines contained in the colostrum and milk of sows are essential for the development of the immune system of piglets, and by activating various lymphocyte subpopulations, they protect neonates against inflammation in the first days after birth.

Stimulation of the Th1 response involves secretion of cytokines IL-2, IFN-γ, and TNF-α, which promote cellular immune mechanisms and ensure the functional response [44]. Among the cytokines released, IL-2 is particularly important. Its effect is manifested as stimulation of the growth of B and T lymphocytes and NK cells and activation of innate immune response mechanisms [44]. In our study, in the group of sows receiving Bokashi preparation, during the entire experimental period, lower concentrations of this cytokine were found in the colostrum and milk as compared to the control. The slight increase in the IL-2 concentration in the milk in the experimental and control groups between 24 and 72 h postpartum may have been due to infection of the mammary gland with bacteria from the animals’ living environment, but is not associated with inflammatory processes in the mammary gland, as clinical examination showed no pathological changes in the mammary gland of the sows (no changes in milk secretion, increase in the temperature of the tissues in the area, redness, swelling, or soreness). In the colostrum of sows receiving Bokashi preparation and in the milk up to 120 h postpartum, a lower IFN-γ level was also observed in comparison with the control. The most likely reason for this is the high concentration of Th2 cytokines, which inhibit the secretion of pro-inflammatory cytokines. The high concentrations of this cytokine in the control group suggest the promotion of a cell-mediated immune response dependent on stimulation of CD4^+^ lymphocytes. The results of the study confirm that IFN-γ inhibits proliferation and activity of Th2 cells and cytokine synthesis by these cells by promoting the pro-inflammatory nature of the immune response. The increasing concentrations of IFN-γ shown in the milk in both groups show that this cytokine plays an important role during lactation by taking part in immune protection of the mammary gland through activation of macrophage phagocytosis or stimulation of cytotoxic (Tc) lymphocytes [43].

In the case of TNF-α and IL-6, higher concentrations of both cytokines were found in the colostrum and milk of sows receiving Bokashi preparation up to 72 h postpartum. TNF-α present in colostrum and milk is secreted by macrophages and the mammary epithelium [45, 46], and a high concentration of this cytokine stimulates development of the mammary gland during colostrogenesis. The TNF-α contained in colostrum and milk has also been shown to inhibit local inflammation of the mammary gland during lactation [47]. The increase in the TNF-α concentration in the experimental group indicates the promotion of cellular immune response mechanisms through enhancement of Th1 lymphocyte activity, which has also been demonstrated in the case of probiotics containing strains of *Leuconostoc* spp. and *Streptococcus* spp. [48]. The high concentration of TNF-α in the colostrum after application of Bokashi preparation may be due to stimulation of systemic immune mechanisms, mainly monocyte and macrophage phagocytosis, by providing the organism with bacterial antigens contained in the probiotic. Stimulation of phagocytosis by TNF-α may result in the release of IL-6 from macrophages, which were present in high concentrations in the colostrum and milk of sows receiving Bokashi preparation. Similar results have been obtained by Blumer et al. [32] who reported high TNF-α expression in the placenta of pregnant mice whose feed was supplemented with *Lactobacillus rhamnosus* GG (LGG). These observations indicate that probiotics induce pro-inflammatory signals activating the cytotoxicity of cells involved in protection against infection. This was confirmed in our study by the increase in TNF-α and IL-6 levels in the colostrum and milk of sows receiving Bokashi preparation in correlation with high serum concentrations of these cytokines.

IL-6 is produced by mammary epithelial cells and mononuclear cells of colostrum and milk [49]. This cytokine affects TNF-α production in the mammary gland, increasing its concentration in the colostrum and milk, as confirmed by the results obtained in the experimental group. IL-6 also influences differentiation of CD4^+^ T lymphocytes towards the Th2 phenotype and secretion of cytokines, mainly IL-4 [50]. This process is associated with impairment of Th1 cell differentiation, but does not directly affect Th2 cells. The high IL-6 concentration in colostrum in conjunction with the high IL-4 concentration in the experimental group suggests that the use of Bokashi preparation influences the differentiation of T lymphocytes towards production of Th2 cytokines. The anti-inflammatory effect of IL-6 consisted in stimulation of IL-10 synthesis, as demonstrated in the experiment. The high concentration of IL-10 in the colostrum of sows receiving Bokashi preparation shows that IL-6 is involved in modulating the immune response and maintaining a balance between Th1 and Th2 cytokine synthesis. Saito et al. [51] showed that IL-6 is also closely linked to local production of IgA in the mammary gland. This is confirmed by the results of our study, in which high concentrations of IL-6 and IgA were noted in the colostrum of the sows from the experimental group.

The use of Bikashi preparation as feed additives for pigs also stimulates secretion of cytokines associated with Th2 lymphocyte activity, mainly IL-4, which activates the humoral immune response. Additional evidence of activation of this type of response is the high IL-4 concentration together with the high levels of IL-10 and TGF-β. The use of Bokashi preparation also stimulates B lymphocytes to produce IgG and IgA antibodies, as demonstrated in the experiment by the high levels of these antibodies in the colostrum and milk. Modulation of IL-4-secreting Th2 cells may lead to changes in the immune response profile towards Th0/Treg or Th1 [50]. The high level of IL-4 in the colostrum of the sows receiving Bokashi preparation in conjunction with the IL-10 concentration indicates that the probiotics had an immunoregulatory effect, which can lead to synthesis of Treg cells maintaining a balance between the Th1 and Th2 response.

One of the most important cytokine markers of the immune response profile is IL-10, which is released by many types of cell, mainly B lymphocytes. The function of IL-10 involves both suppression of the immune response and modulation of Th cells [52]. In vitro studies have shown that IL-10 inhibits the synthesis and release of pro-inflammatory cytokines, including IL-1, TNFα, IL-6, and IL-12, by antigen-presenting cells [53, 54] and inhibits T lymphocyte proliferation in response to an antigen or superantigen. In the present study, in the colostrum and milk of the pigs receiving Bokashi preparation, a higher IL-10 concentration was observed in comparison to the control up to 72 h postpartum. Similarly, a higher concentration of this cytokine was noted in the stimulated colostrum PMN cells. The high IL-10 concentration shown in the study following the use of Bokashi preparation, in correlation with the high IL-4 and low IFN-γ levels, suggests dominance of the Th2 immune profile, a reduction in the intensity of the inflammatory process, enhancement of the humoral immune response, and at the same time inhibition of Th1 cytokine production.

An important anti-inflammatory and immunoregulatory cytokine is TGF-β. In the sows receiving Bokashi preparation, a high concentration of this cytokine was noted in the serum on day 114 of gestation, in the colostrum and milk postpartum, and also in the stimulated colostrum PMC cells. These phenomena are indicative of suppression of the inflammatory immune response, inhibition of the Th1 response, and promotion of the Th2 response. Similar observations have been made in humans by Isolauri et al. [55], who demonstrated that the use of *Lactobacillus rhamnosus* as a dietary supplement for 4 weeks before birth resulted in an increase in TGF-β and IgA concentrations in milk, and additionally suppressed allergic reactions in neonates. Research on an animal model has also confirmed the efficacy of probiotics as agents increasing the TGF-β concentration in colostrum and milk and participating in the development of tolerance to food allergens [45]. In this context, it is important that Bokashi preparation can be used in the diet of sows to ensure food tolerance in the first days of life of piglets, i.e., during the colostrum period.

TGF-β also has a regulatory effect on the immune response by facilitating differentiation of activated B and T lymphocytes into a regulatory T cell population [56]. The high concentrations of pro-inflammatory cytokines IL-2 and TNF-α observed at 114 days of gestation and of IFN-γ on days 60 and 114, in correlation with the high TGF-β level, are indicative of its regulatory effect. This effect results in maintenance of homeostasis in the body and suppression of the inflammatory immune response, which is a reaction to developing fetuses and colonization of the gastrointestinal tract by probiotic microorganisms.

TGF-β also promotes synthesis of antibodies by B lymphocytes, mainly IgA antibodies, which protect the intestinal mucosa against damage [57], as demonstrated in the experiment. The high levels of IgG and IgA noted in the colostrum and milk indicate a protective role of mammary gland secretion in the first few days of life of the piglets. The initial decrease in the IgA concentration in the milk in the experimental group, followed by an increase starting at 48 h postpartum, in correlation with the decrease in the IgG concentration, indicates a switch from IgG to IgA mediated by TGF-β in co-stimulation with IL-10. The higher concentration of IgA antibodies in the colostrum and milk is important in the context of preventing antigen adhesion to enterocytes in the intestines of the piglets and reducing the risk of infection and death [58]. Similarly, the higher serum IgA concentration noted in the sows in our study indicates more effective immune protection of the pregnant female.

In addition to its nutritional value, colostrum is an important source of immunoglobulins ensuring passive protection for sucklings. The IgG and IgA antibodies in colostrum are involved in systemic and local defense processes in the first days of life of piglets until the GALT system is activated [59]. The results of our study indicate that the use of Bokashi preparation in sows enhances the immune potential of the colostrum by increasing the concentrations of IgG and IgA. Similar results were obtained by Vitini et al. [60] and Valeur et al. [61], who demonstrated an increase in IgA concentration in the intestinal mucosa of pigs following dietary supplementation with *Bifidobacterium longum* and *S. boulardii*, which influence the systemic immune response. The immunopotentiation demonstrated in the present study should be considered to be linked to activation by the probiotics of B lymphocyte subpopulations and Th2 cytokines, mainly IL-4, which enter the body fluids, including colostrum and milk [62, 63]. A high concentration of immunoglobulins in colostrum and milk ensures piglets passive humoral immunity through antigen agglutination, neutralization of viruses and bacterial toxins, and prevention of bacterial adhesion to epithelial cells, and protects the mammary gland against infection through antigen binding and participation in immune processes.

The immunomodulatory function of Bokashi preparation is also confirmed by the high concentrations of Th1 and Th2 cytokines observed in the culture of stimulated colostrum PMC cells. The low concentration of IL-2 in the absence of differences in IFN-γ concentrations between the experimental and control groups confirms the anti-inflammatory effect of Bokashi preparation on pigs. The induction of immunosuppressive cytokines, mainly IL-10, noted in the colostrum PMCs may be linked to the ‘bystander suppression’ process, which leads to suppression of the pro-inflammatory response by reducing dendritic cell activation [64]. This phenomenon and the accompanying down-regulation of TNFα show that the use of Bokashi preparation in pig diets ensure a balance between the Th1 and Th2 response in the body [64, 65].

The immunological effects of probiotics largely depend on the type of microorganisms they are composed of. For example, *Lactobacillus rhamnosus* and *Bacillus lactis* have been shown to increase the concentration of immunoglobulins and cytokines in milk, although probiotics based on *B. lactis* alone have a more favorable effect on this process [33]. Significant differences in the properties of probiotic strains were recently demonstrated by Bottcher et al. [53] who showed that dietary supplementation with *Lactobacillus reuteri* resulted in a decrease in the TGF-β2 concentration in human milk without affecting the concentration of IgA, TGF-β1, and other cytokines. In this context, the use of Bokashi preparation as feed additives for pigs seems to be beneficial, as it is composed of a variety of microbes acting synergistically, which ensures an increase in the immune potential of colostrum through simultaneous stimulation of the Th1 and Th2 responses.

It is worth emphasizing that the use of Bokashi preparation during pregnancy and lactation had a positive effect on the health of the sows. During the experiment, sow body weight at parturition was higher and weight loss during the first 7 days of lactation was lower. No significant differences were noted in feed consumption between the sows in the control and experimental groups. Mori et al. [66] and Klingspor et al. [67] showed that probiotics increase the capacity for nutrient absorption by the intestinal mucosa in pigs, while Alexopoulos et al. [19] found that these substances increase serum levels of certain nutrients, such as cholesterol and fats. The results of these studies together with those obtained in our experiment suggest that the use of Bokashi preparation in sows may improve the digestibility of dietary nutrients owing to the increased production and activity of digestive enzymes in the gut induced by the microbial strains contained in the supplement. These processes enhance digestive processes in the gut. Furthermore, the use of EM Bokashi improved the apparent total tract digestibility of nitrogen and energy, which requires further study. Better utilization of nutrients during pregnancy and lactation directly affects colostrum and milk quality, which is confirmed by the results of the study. The high immune status of the colostrum and milk of the sows whose diet was supplemented with Bokashi preparation significantly reduced piglet mortality in the first 7 days of life and the percentage of piglets with symptoms of diarrhea. Effective protection of the intestines against infection by diarrhea-inducing pathogens, owing to high levels of immunoglobulins and cytokines in the colostrum, leads to better performance results and reduces financial losses on pig farms [19, 68, 69].

Interest in the use of probiotics during pregnancy and lactation in both humans and animals has grown significantly with the appearance of the ‘hygiene hypothesis’ [70]. Early exposure of the female to probiotic microorganisms has proven to have a significant effect on the programming of the neonatal immune system [71]. Studies on humans have shown that the use of probiotics during pregnancy affects the composition of the colostrum and milk, protecting the offspring against the development of allergic diseases [72, 73]. Similarly, animal studies have shown that supplementation of feed with probiotics during pregnancy inhibits the development of respiratory disease in the offspring, which is evidence of their immunomodulatory effect on the fetus [74, 75]. We observed a similar effect after using Bokashi preparation in the diet of pregnant sows. The use of Bokashi preparation as feed additives also resulted in increased concentrations of pro-inflammatory cytokines TNF-α and IL-6, which increase the protective capacity of the colostrum by stimulating cellular immune mechanisms protecting the mother and neonates against infection. At the same time, the increased concentrations of cytokines IL-4, IL-10, TGF-β, and of immunoglobulins in the colostrum and milk from sows in the experimental group demonstrate the immunoregulatory effect of Bokashi preparation on Th2 cells and may lead to increased expression of regulatory T cells and polarization of the immune response from Th1 to Th2.
